# Core factor of NEXT complex, ZCCHC8, governs the silencing of LINE1 during spermatogenesis

**DOI:** 10.1093/nsr/nwae407

**Published:** 2024-12-17

**Authors:** Rushuang Yan, Meijie Qi, Pengfei Zhang, Bin Shen, Jiqing Yin, Chuan Chen, Silin Tian, Lin Chen, Xingxu Huang, Hong Wang, Shaorong Gao, You Wu, Yawei Gao

**Affiliations:** State Key Laboratory of Cardiology and Medical Innovation Center, Department of Reproductive Medicine Center, Shanghai East Hospital, Frontier Science Center for Stem Cell Research, School of Life Sciences and Technology, Tongji University, Shanghai 200092, China; Clinical and Translation Research Center of Shanghai First Maternity & Infant Hospital, Frontier Science Center for Stem Cell Research, School of Life Sciences and Technology, Tongji University, Shanghai 200092, China; Reproductive and Genetic Center, The First Affiliated Hospital of USTC, Division of Life Sciences and Medicine, University of Science and Technology of China, Hefei 230001, China; State Key Laboratory of Reproductive Medicine and Offspring Health, Women's Hospital of Nanjing Medical University, Nanjing Women and Children's Healthcare Hospital, Gusu School, Nanjing Medical University, Nanjing 211166, China; Zhejiang Lab, Hangzhou 311121, China; State Key Laboratory of Reproductive Medicine and Offspring Health, Women's Hospital of Nanjing Medical University, Nanjing Women and Children's Healthcare Hospital, Gusu School, Nanjing Medical University, Nanjing 211166, China; State Key Laboratory of Cardiology and Medical Innovation Center, Department of Reproductive Medicine Center, Shanghai East Hospital, Frontier Science Center for Stem Cell Research, School of Life Sciences and Technology, Tongji University, Shanghai 200092, China; State Key Laboratory of Cardiology and Medical Innovation Center, Department of Reproductive Medicine Center, Shanghai East Hospital, Frontier Science Center for Stem Cell Research, School of Life Sciences and Technology, Tongji University, Shanghai 200092, China; Women's Hospital, Zhejiang University School of Medicine, Hangzhou 310006, China; State Key Laboratory of Cardiology and Medical Innovation Center, Department of Reproductive Medicine Center, Shanghai East Hospital, Frontier Science Center for Stem Cell Research, School of Life Sciences and Technology, Tongji University, Shanghai 200092, China; State Key Laboratory of Cardiology and Medical Innovation Center, Department of Reproductive Medicine Center, Shanghai East Hospital, Frontier Science Center for Stem Cell Research, School of Life Sciences and Technology, Tongji University, Shanghai 200092, China; Zhejiang Lab, Hangzhou 311121, China; State Key Laboratory of Cardiology and Medical Innovation Center, Department of Reproductive Medicine Center, Shanghai East Hospital, Frontier Science Center for Stem Cell Research, School of Life Sciences and Technology, Tongji University, Shanghai 200092, China; State Key Laboratory of Cardiology and Medical Innovation Center, Department of Reproductive Medicine Center, Shanghai East Hospital, Frontier Science Center for Stem Cell Research, School of Life Sciences and Technology, Tongji University, Shanghai 200092, China; Clinical and Translation Research Center of Shanghai First Maternity & Infant Hospital, Frontier Science Center for Stem Cell Research, School of Life Sciences and Technology, Tongji University, Shanghai 200092, China; Shanghai Institute of Stem Cell Research and Clinical Translation, Shanghai 200120, China; State Key Laboratory of Cardiology and Medical Innovation Center, Department of Reproductive Medicine Center, Shanghai East Hospital, Frontier Science Center for Stem Cell Research, School of Life Sciences and Technology, Tongji University, Shanghai 200092, China; Clinical and Translation Research Center of Shanghai First Maternity & Infant Hospital, Frontier Science Center for Stem Cell Research, School of Life Sciences and Technology, Tongji University, Shanghai 200092, China; Shanghai Institute of Stem Cell Research and Clinical Translation, Shanghai 200120, China; State Key Laboratory of Cardiology and Medical Innovation Center, Department of Reproductive Medicine Center, Shanghai East Hospital, Frontier Science Center for Stem Cell Research, School of Life Sciences and Technology, Tongji University, Shanghai 200092, China; Shanghai Institute of Stem Cell Research and Clinical Translation, Shanghai 200120, China

**Keywords:** transposon elements, nuclear exosome targeting complex, LINE1, spermatogonial stem cells, spermatogenesis

## Abstract

The overactivation of transposable elements (TEs) is a significant threat to male reproduction, particularly during the delicate process of spermatogenesis. Here, we report that zinc finger protein ZCCHC8—a key component of the nuclear exosome targeting (NEXT) complex that is involved in ribonucleic acid (RNA) surveillance—is required for TE silencing during spermatogenesis. Loss of ZCCHC8 results in delayed meiotic progression and reduced production of round spermatids (RS). We observed that young long-interspersed nuclear element (LINE1, L1) subfamilies that are targeted by ZCCHC8 were upregulated in both spermatogonial stem cells (SSC) and pachytene spermatocytes (PS) of *Zcchc8* null testes. Further study found that a reduced H3K9me3 modification in SSC and elevated H3 lysine 4 trimethylation in the PS of *Zcchc8* KO mice occurred upon young L1, especially L1Md_A, which may have contributed to impairment of the chromatin condensation from PS to RS during spermatogenesis. This study highlights the crucial role of RNA surveillance-mediated chromatin repression by the NEXT complex during spermatogenesis.

## INTRODUCTION

Transposable elements (TEs) comprise a significant portion of the mammalian genome [1,2] and some of them present a threat to genomic integrity through their replicative mechanism, which can produce insertions that disrupt the expression of adjacent genes [2–4]. Meanwhile, TEs can significantly influence gene transcription and the status of chromatin [5,6]. Notably, in male mouse germ cells, excessive TE activation is consistently linked with meiotic failure, azoospermia and male sterility [7–9]. During spermatogenesis, the activity of TEs, such as that of long-interspersed nuclear element (LINE1, L1), is tightly regulated through various mechanisms, which include *de novo* DNA methylation, repressive histone modifications such as H3K9me2/3 and post-transcriptional or transcriptional silencing that is mediated by PIWI proteins and their associated piRNAs [7,8,10–17]. However, a comprehensive understanding of the post-transcriptional regulatory mechanisms of TEs remains elusive, signifying a crucial area for further research.

In eukaryotic cells, the ribonucleic acid (RNA) exosome, which is a complex 3′>5′ ribonuclease, is essential for RNA degradation and quality control, which is guided by adaptors: the nuclear exosome targeting (NEXT) complex and the pA-tail exosome targeting (PAXT) complex in the nucleus [18–22]. The NEXT complex comprises the RNA recognition motif (RRM)-containing protein RBM7, the Zn-knuckle protein ZCCHC8 and the RNA helicase hMTR4/SKIV2L2 in mammalian cells. The NEXT complex is responsible for targeting and degrading a variety of RNA substrates, including promoter upstream transcripts (PROMPTs), enhancer RNAs (eRNAs), transposable element RNAs (TE RNAs) and specific long non-coding RNAs (lncRNAs) [23–26]. Mutations in ZCCHC8 are linked to familial pulmonary fibrosis and ZCCHC8 knockout in mice alters telomerase maturation, affecting its RNA component [27]. Additionally, mutations in RBM7 are associated with spinal motor neuropathy in human diseases [24,28]. Our previous study showed that *Zcchc8* KO mice exhibited reduced fertility rates [24]. However, the specific impact of the NEXT complex on male fertility and spermatogenesis warrants further investigation.

ZCCHC8, which is the core factor of the NEXT complex, has also been implicated in influencing chromatin states. In our previous work, we demonstrated that the NEXT complex degrades the L1 transcripts, thereby influencing chromatin openness during early embryonic development [24]. More recently, the NEXT complex has been observed to interact with the m^6^A reader YTHDC1, potentially facilitating PROMPT, eRNA and TE RNA degradation, and may also influence the chromatin accessibility of adjacent genes [29,30]. In embryonic stem cells, the knocking-out of ZCCHC8 resulted in elevated TE RNA levels, as reported in both our study and that of Garland W *et al.*, likely due to impaired RNA decay and the interaction of ZCCHC8 with the human silencing hub (HUSH) complex. This interaction is maintained at TE loci, which are recruited by SETDB1, thereby supporting the maintenance of H3K9me3 modifications [25]. Thus, unraveling the influence of ZCCHC8 on chromatin states is crucial for understanding its regulatory mechanisms in meiosis.

In this study, we employed *Zcchc8* knockout and tagged mouse models to analyse its impact on chromatin states. Our results reveal that ZCCHC8 directly binds to retrotransposon transcripts—particularly to young L1 elements during spermatogenesis. Knockout of ZCCHC8 not only upregulates their RNA levels, but also alters chromatin states in specific regions, characterized by a decrease in H3K9me3 and an increase in H3 lysine 4 trimethylation (H3K4me3). These changes lead to chromatin decondensation and anomalies in the transition from spermatocytes to round spermatids (RS), ultimately resulting in aberrant gametogenesis. In this study, we propose that the NEXT complex serves as a crucial nuclear RNA decay factor that regulates retrotransposons at both RNA and epigenetic levels, which uncovers a new mechanism of retrotransposon silencing during spermatogenesis.

## RESULTS

### Reproduction defects in *Zcchc8* KO male mice

**Figure 1.**
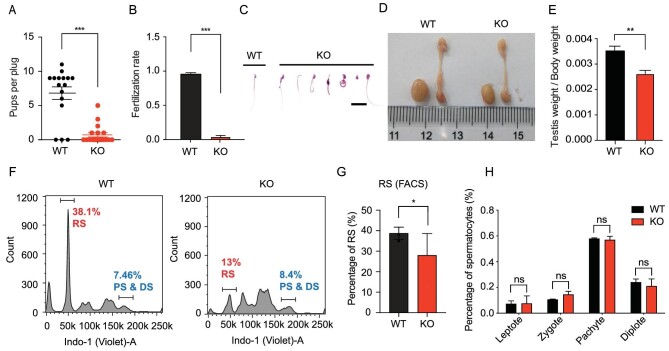
Defects of gametogenesis in *Zcchc8* KO mice. (A) Litter size from WT and *Zcchc8* KO adult male mice crossed with WT female mice. Each plot represents a plug after successful mating. (B) Average fertilization rate after *in vitro* fertilization (IVF) using WT MII oocytes and *Zcchc8* control and KO sperm. *N* = 3. (C) Statistical analysis of sperm density in adult epididymis of *Zcchc8* WT and KO mice. *N* = 3. (D) Photo of testis and epididymis of WT and *Zcchc8* KO adult mice. (E) Bar plots showing weight ratio of testis versus body of WT and *Zcchc8* KO adult mice. *N* = 5. (F) Cell count and ratio of WT and *Zcchc8* KO adult testis sorted by FACS. PS, pachytene spermatocytes; DS, diplotene spermatocytes; RS, round spermatids. (G) Bar plots showing percentage of RS counted by FACS. *N* = 4. (H) Percentage of spermatocytes calculated through chromatin spread and immunostaining of γH2A.X and SYCP3. *n* = 200 (*n*, spermatocytes were counted for each testis). Data in (A–C), (E) and (G) are presented as the mean ± SEM of biological replicates. Unpaired one-tailed Student's *t*-test was used to calculate the *P*-values in (A)–(C), (E) and (G).*P < 0.05; **P < 0.01; ***P < 0.001. NS, not significant.

As mentioned in previous study, we engineered *Zcchc8* KO mice by utilizing the CRISPR/Cas9 technology and reported the male subfertility upon ZCCHC8 depletion [24] (Fig. 1A and Fig. S1A–C). These findings revealed that ZCCHC8 may play a regulatory role in gametogenesis, which is supported by the specific expression pattern of ZCCHC8. We found that, at the spermatogonial stage, ZCCHC8 expression was observed in all cell types within postnatal day 6 (P6) testis (Fig. S1D). However, in adult mice, ZCCHC8 exhibited moderate expression levels in spermatocytes and was significantly upregulated in round and elongating spermatids, yet it was absent in elongated spermatids (Fig. S1E and F). *In vitro* fertilization assays revealed a drastically reduced fertilization rate of <5% when *Zcchc8* KO sperm were used with wild type (WT) oocytes (Fig. 1B). Further analysis showed that the epididymis of ZCCHC8 KO mice exhibited a significantly reduced sperm count, increased sperm deformities and decreased sperm motility (Fig. 1C and Fig. S2A–E). Additionally, Zcc*hc8* KO mice exhibited a significant reduction in testes/body weight ratio (Fig. 1D and E). To further clarify the defects in spermatogenesis, we analysed cell proportions in the adult testis by using Hoechst staining and cell sorting by fluorescence-activated cell sorting (FACS). We found that the KO mice exhibited a significant reduction in the population of RS in the testes (Fig. 1F and G). However, all stages of spermatogenesis in the seminiferous tubules appeared to be normal without any obvious stage-specific block (Fig. S2F and G, and Table S1). Furthermore, there was an observed increase in apoptotic cells in the *Zcchc8* KO testes (Fig. S2H–J). These results suggest that disturbances occur during the meiosis from spermatocytes to RS—a critical transitional phase of meiotic exit and chromatin compaction. Subsequent analysis showed that the spermatocyte ratios, as assessed by using chromosome spreads and immunofluorescence, were similar between KO and WT testes in the first meiotic prophase, indicating that defects occur after the diplotene stage in the progression to RS (Fig. 1H and Fig. S2K). Collectively, these observations indicate that ZCCHC8 depletion impairs the meiotic process, leading to a reduction in RS production and increased apoptosis in testicular cells.

### The first wave of spermatogenesis was delayed in *Zcchc8* KO mice

**Figure 2.**
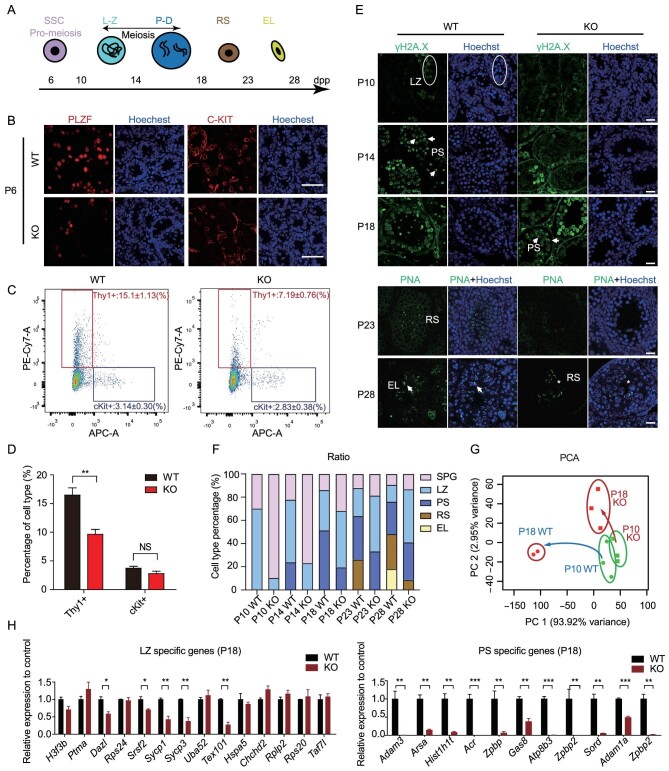
First wave of spermatogenesis was delayed in *Zcchc8* KO mice. (A) Schematic diagram of the first wave of spermatogenesis. (B) Immunostaining of PLZF and c-KIT in P6 testes of WT and *Zcchc8* KO mice. Scale bar = 50 µm. (C) FACS results of P6 testicular cells marked by THY1 and c-KIT antibodies of *Zcchc8* WT and KO mice. (D) Percentage of cell type of THY1+ and c-KIT+ cells in WT and *Zcchc8* KO mice. *N* = 3. (E) Appearance time of L–Z, PS and RS in WT and *Zcchc8* KO mice. Immunostaining of γH2A.X and peanut agglutinin (PNA) are markers for PS and RS. Scale bar = 20 µm. L–Z, leptotene–zygotene spermatocytes; PS, pachytene spermatocytes; RS, round spermatids; EL, elongated spermatids. (F) Percentage of spermatocytes calculated through immunostaining of γH2A.X and PNA in P10, P14, P18 and P28 testes of *Zcchc8* WT and KO mice. Data are presented as the mean of three biological replicates. Raw data of cell numbers counted in each biological replicate are shown in Table S2. (G) Principle component analysis of RNA-seq showing transcriptome difference of WT and *Zcchc8* KO testes of P10 and P18. (H) Relative expression level of L–Z and PS specific genes measured by RNA-seq of P18 testes. *N* = 2. Data in (D) and (H) are presented as the mean ± SEM of biological replicates. Unpaired one-tailed Student's *t*-test was used to calculate the *P*-values in (D) and (H). **P < 0.01; ***P < 0.001.

In consideration of the diminished production of RS, we sought to examine the first wave of spermatogenesis from P6 to P28 (Fig. 2A). At the age of P6–P8, which is the initial stage of spermatogenesis, which contains undifferentiated spermatogonia with self-renewing ability (marked by PLZF+ or THY1+) and c-KIT+ differentiating spermatogonia that are committed to gametogenesis (marked by c-KIT+) [14], *Zcchc8* KO mice exhibited a significantly reduced number of undifferentiated spermatogonia (THY1+) but the population of differentiated spermatogonia (c-KIT+) did not show a significant reduction (Fig. 2B–D). Transcriptional analysis revealed a greater number of differentially expressed genes (DEGs) in the THY1+ cells (581 upregulated and 399 downregulated genes) compared with the c-KIT+ cells (217 upregulated and 314 downregulated genes) (Fig. S3A). Gene ontology (GO) analysis indicated that genes related to male meiosis were downregulated in THY1+ cells (Fig. S3B and C). This analysis underscores the impact of ZCCHC8 deficiency in the initial phase of spermatogenesis, particularly affecting the population and genetic expression of spermatogonial stem cells (SSC).

We then analysed four representative stages of first spermatogenesis that included the meiotic prophase I leptotene–zygotene (L–Z) stage at P10, the pachytene–diplotene (P–D) stage at P14–P18, RS at P23 and elongated spermatids (EL) at P28. Through labeling with γH2A.X and Hoechst33342, a delayed progression of meiosis was observed in KO mice, as evidenced by an obvious postponed emergence of pachytene spermatocytes (PS) and RS (Fig. 2E and F, and Table S2). We then focused on two representative times of meiosis prophase I stages, P10 and P18, for further analysis. The testis weights of ZCCHC8 KO mice at P10 and P18 were significantly lower than those of WT mice (Fig. S3D). Notably, the transcriptional profile of KO testes at P18 more closely resembled that of WT testes at P10 according to principal component analysis (PCA) (Fig. 2G), which is consistent with histological results. The number of DEGs in KO testes was approximately four times higher at P18 (3258 DEGs) than at P10 (825 DEGs), suggesting escalating defects and developmental delays during the first meiotic prophase (Fig. S3E). Further GO analysis of these DEGs revealed that genes related to DNA packaging were downregulated at P10, while genes associated with meiosis and spermatogenesis were downregulated at P18 (Fig. S3F and G). Notably, over half of the genes that were specific to the L–Z stages retained normal expression levels in *Zcchc8* KO P18 testes, while the vast majority of genes that were exclusive to the pachytene stage were significantly downregulated at P18 (Fig. 2H).

In summary, these findings demonstrate that *Zcchc8* KO mice exhibit a delay of the first wave of spermatogenesis, with significant disruptions in gene expression and meiotic progression.

### Young L1 elements were elevated in *Zcchc8* KO SSC and spermatocytes.

We previously reported that ZCCHC8 can bind to TE RNA, especially young L1 RNA in embryonic stem cells (ESCs) and early embryos, leading to an increase in L1 and other repeat RNA levels, as well as enhanced chromatin accessibility [24]. This prompted us to investigate whether ZCCHC8 also regulates transposable elements during spermatogenesis. Here, we initially examined the TE RNA expression levels in the first wave of spermatogenesis. Our analysis revealed that several TEs, including LINE1, IAP, MERVL and MusD/Etn, were upregulated in the THY+ SSC and P18 testes of KO mice, but with no significant increase in c-KIT+ spermatogonia and P10 testes (Fig. S4A).

**Figure 3.**
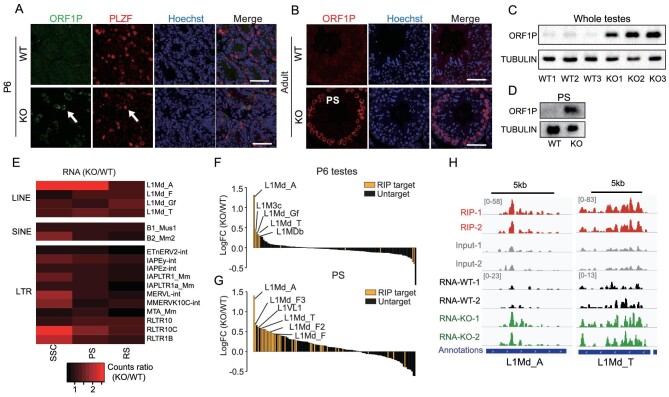
ZCCHC8 targeted young L1 was overactivated in SSC and PS in KO mice. (A) Immunostaining of ORF1P and PLZF in P6 testes of WT and *Zcchc8* KO mice. Arrows indicate SSC with positive signal of PLZF and ORF1P. Scale bar = 50 μm. ORF1P antibody: homemade. (B) Immunostaining of ORF1P in adult testes of WT and *Zcchc8* KO mice. Positive ORF1P cells are PS cells. Scale bar = 50 μm. ORF1P antibody: homemade. (C) Western blot of ORF1P and TUBULIN in WT and *Zcchc8* KO adult whole testes. ORF1P antibody: Abcam. (D) Western blot of ORF1P and TUBULIN in WT and *Zcchc8* KO adult PS cells. ORF1P antibody: Abcam. (E) Heat map showing RNA fold change in KO/WT SSC, PS and RS. Representative and highly expressed repeat subfamilies are listed. Data are presented as the mean of two biological replicates. (F) RNA logFC (KO/WT) of all L1 subfamilies are represented by a bar plot. Yong L1 subfamilies are marked. Data are presented as the mean of two biological replicates. (G) RNA logFC (KO/WT) of all L1 subfamilies are represented by a bar plot. Yong L1 subfamilies are marked. Data are presented as the mean of two biological replicates. (H) Genome browser track showing RIP and RNA-seq signal of L1Md_A and L1Md_T in PS.

Immunostaining for LINE1 ORF1 protein (ORF1p) revealed obvious elevated levels uniquely in *Zcchc8*-depleted SSC (PLZF+ cells) and in PS in adult testes, confirmed by using Western blot analysis of whole testes and isolated PS (Fig. 3A–D and Fig. S4B). Furthermore, ORF1P upregulation was also observed as early as in P0 germ cells (DDX4+ cells) and in PS during the first wave of spermatogenesis at P18 in the absence of ZCCHC8 (Fig. S4C and D).

To confirm that the increase in L1 RNA was obtained in adult mice, we isolated PS and RS from adult testes for RNA-seq and qPCR. Consistently with earlier observations, L1 and other repetitive elements such as IAP, MERVL and MusD/ETn were significantly upregulated in PS cells but not in RS cells (Fig. S4E). Further analysis of RNA-seq data revealed that L1Md_A, which is a prominent member of the L1 subfamily, was the TE most upregulated in KO SSC and PS cells but negligible differences were exhibited in RS cells (Fig. 3E and Fig. S4F). Given the notably lower RS proportion in *Zcchc8* KO mice, it is conceivable that the elevated transposon RNA in *Zcchc8* KO mice may have impeded the transition from PS to RS.

In previous studies, the piRNA pathway was indispensable for TE silencing through post-transcriptional gene silencing and DNA methylation direction [31]. We then wondered whether ZCCHC8 KO would trigger the defects in piRNA generation. We therefore sequenced small RNA from P0 testes and PS cells that were sorted from adult testes to analyse piRNA biogenesis in WT and KO mice. We found no significant changes in piRNA (length = 26 nt) in P0 testes and adult PS cells (Fig. S5A and B, and Table S3). Additionally, the proportion of piRNAs that were derived from LINE elements did not show a significant difference. At the subfamily level, L1Md_A and L1Md_T also remained unchanged in both P0 testes and adult PS cells (Fig. S5C–F). Additionally, we examined the piRNA-related proteins such as MILI, MIWI and TDRKH, which were expressed normally in Zcchc8 KO testes (Fig. S5G). These results suggested that ZCCHC8 regulates L1 silencing independently of the piRNA pathway.

### ZCCHC8 targeted young L1 transcripts in SSC and PS

To explore whether ZCCHC8 directly targets these transposon transcripts, we employed RNA immunoprecipitation sequencing (RIP-seq) to analyse the binding targets of ZCCHC8 during spermatogenesis. Due to poor immunoprecipitation (IP) efficiency with ZCCHC8 antibodies, we generated a mouse model with an endogenously tagged ZCCHC8 protein for enhanced detection (Fig. S6A). For SSC, which are limited by cell numbers, we utilized whole testes of P6 mice for full-length RIP-seq. Reads distribution analysis revealed that ZCCHC8 can bind LINE RNAs, though with moderate significance (Fig. S6B and C). Further examination of L1 subfamilies among RIP targets showed that most upregulated L1 elements are indeed targeted by ZCCHC8 (Fig. 3F). In adult testes, upon isolating PS, we observed a selective affinity of ZCCHC8 for L1 transcripts (Fig. S6D and E), corroborating that the majority of elevated L1 subfamilies are indeed targeted by ZCCHC8 (Fig. 3G). In both P6 testes and PS cells, the significantly upregulated ZCCHC8-targeted L1 transcripts predominantly belong to young L1, including L1Md_A, L1Md_T and L1Md_Gf (Fig. 3H and Fig. S6F and G). These results suggest that ZCCHC8 plays a crucial role in repressing young L1 subfamilies through directly targeting and influencing their levels.

### ZCCHC8 deletion resulted in H3K9me3 loss on young L1 elements in SSC

**Figure 4.**
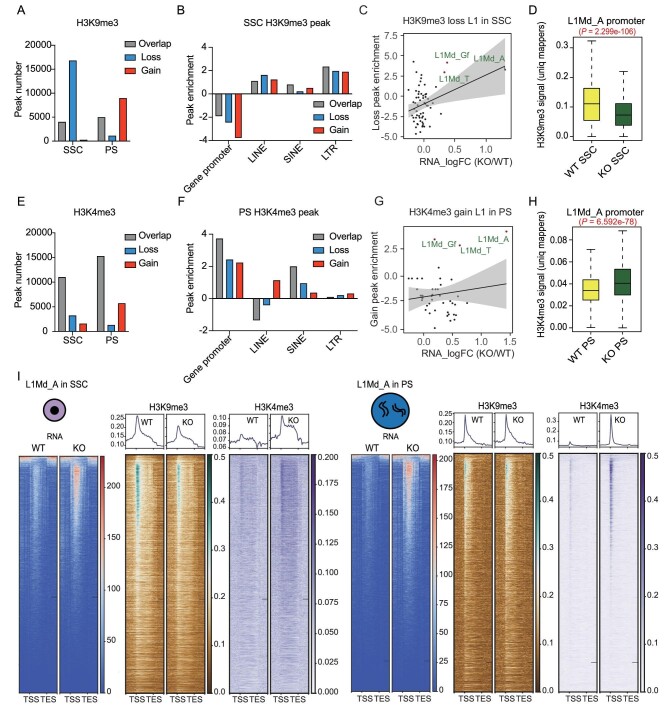
Histone modifications of H3K9me3 and H3K4me3 in SSC and PS. (A) H3K9me3 peak number of gain, loss and overlapping peaks based on KO vs WT in SSC and PS. Data are presented using pooled samples with three replicates (see ‘Methods’). (B) H3K9me3 peak enrichment of gain, loss and overlapping peaks in different genomic regions in SSC. Data are presented using pooled samples with three replicates (see ‘Methods’). (C) H3K9me3 loss peak enrichment of L1 subfamilies and RNA logFC (KO/WT) in SSC. (D) Boxplot showing H3K9me3 signal intensity at promoter regions (–3 to +3 kb of the transcription start site (TSS)) of intact L1Md_A (L1Md_A length > 3 kb, *n* = 3958) loci in SSC, calculated using unique mappers. *P* = *P*-value calculated by Wilcoxon test, two-sided. (E) H3K4me3 peak number of gain, loss and overlapping peaks based on KO vs WT in SSC and PS. Data are presented using pooled samples with three replicates (see ‘Methods’). (F) H3K4me3 peak enrichment of gain, loss and overlapping peaks in different genomic regions in PS. (G) H3K4me3 loss peak enrichment of L1 subfamilies and RNA logFC (KO/WT) in PS. (H) Boxplot showing H3K4me3 signal intensity at promoter regions (–3 to +3 kb of TSS) of intact L1Md_A (L1Md_A length > 3 kb, *n* = 3958) loci in PS, calculated using unique mappers. *P* = *P*-value calculated by Wilcoxon test, two-sided. (I) Heat map of RNA, H3K9me3 and H3K4me3 signal, and average profile of histone modifications of all L1Md_A loci ordered by average RNA level in WT.

Previous research indicated that the NEXT complex collaborates with the HUSH complex to regulate TE RNA expression and establish H3 lysine 9 trimethylation (H3K9me3) modifications in ESCs [25]. Consequently, we investigated whether ZCCHC8 affects the H3K9me3 on retrotransposons during spermatogenesis. We performed native chromatin immunoprecipitation (N-ChIP) sequences for the histone modification H3K9me3 in sorted THY+ SSC at P6 and PS cells in adult mice. Overall, the Pearson correlation indicated a high degree of correlation between biological replicates (Fig. S7A). H3K9me3 peaks showed an obvious reduction in KO SSC but an increase in KO PS, with a similar distribution of peaks across genomic regions (Fig. S7B and C). We analysed the gain and loss of peaks in KO versus WT, finding 16 787 loss peaks in SSC that were primarily enriched in LINE elements (Fig. 4A and B). Notably, young L1 subfamilies such as L1Md_A, L1Md_T and L1Md_Gf exhibited a high degree of peak enrichment, suggesting that the upregulation of these TEs may be due to the loss of H3K9me3 (Fig. 4C and D, and Fig. S7D). Interestingly, we found that, in PS, the H3K9me3 peak gained in LINE and long terminal repeat (LTR) (Fig. S7E) and slightly increased at L1Md_A in KO PS (Fig. S7F and G). Therefore, ZCCHC8 is crucial for the regulation of H3K9me3 modifications on retrotransposons in SSC; as meiosis progresses, other factors may be involved in the establishment of H3K9me3 and compensate for the deficiencies.

### ZCCHC8 KO leads to increased H3K4me3 at young L1 elements

In the previous research on the effects of DNMT3L and MIWI2 double knockout on spermatogenesis, it was found that the absence of DNA methylation during meiosis leads to an increase in H3K4me3 modifications on TEs, causing problems with meiotic recombination [32]. We wondered whether anomalies in H3K4me3 modifications occur at young L1 elements in *Zcchc8* KO spermatocytes. We analysed the H3K4me3 in SSC and PS, with all replicates highly comparable, and found no dramatic change in the H3K4me3 peak number, but there was a notable increase in the peak ratio in distal intergenic regions from SSC to the PS stage (Fig. S8A–C). Further classification of differential peaks between KO and WT revealed that there were 5684 KO-gained peaks enriched on LINE elements and the overlapping peaks were mainly enriched on short interspersed nuclear element (SINE) elements in PS (Fig. 4E and F) but not in SSC (Fig. S8D). Among the LINE subfamilies, L1Md_A, L1Md_T and L1Md_Gf showed a high degree of peak enrichment in PS (Fig. 4G). Upon further analysis of the signal profile and intensity on L1Md_A, we found a significant increase in the L1Md_A promoter region in both SSC and PS, but the overall signal was upregulated more drastically in PS compared with SSC (Fig. 4H and Fig. S8E–H). We further scrutinized all genomic loci of L1Md_A, ordered by the RNA expression levels, and observed a consistent pattern of H3K9me3 loss in SSC and H3K4me3 gain in PS (Fig. 4I).

### Higher chromatin accessibility at L1Md_A in the absence of ZCCHC8 in spermatocytes and spermatids.

**Figure 5.**
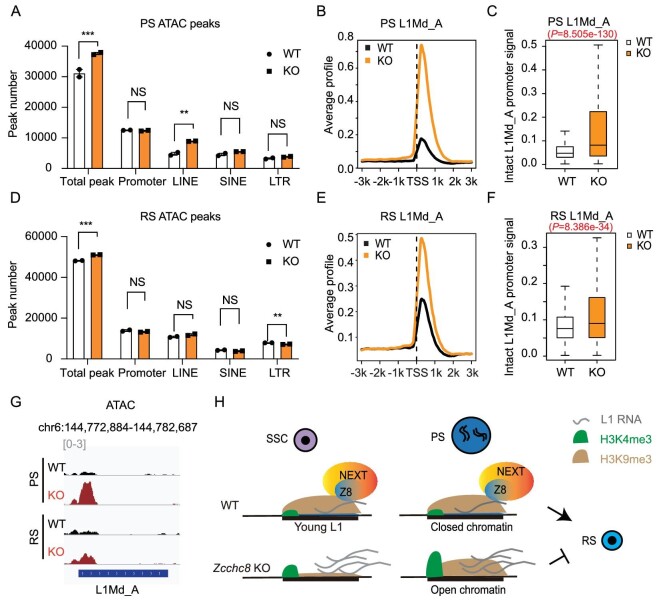
Higher chromatin accessibility at young L1 in KO PS and RS. (A) Peak number of ATAC-seq in different genomic regions in PS. *N* = 2. (B) Average signal profile of ATAC at intact L1Md_A promoters (L1Md_A length > 3 kb, *n* = 3958) in PS. (C) Boxplot showing ATAC signal intensity at intact L1Md_A promoters in PS. (D) Peak number of ATAC-seq in different genomic regions in RS. *N* = 2. (E) Average signal profile of ATAC at intact L1Md_A promoters in RS. (F) Boxplot showing ATAC signal intensity at intact L1Md_A promoters in RS. (G) Genome browser track showing ATAC signal of a L1Md_A locus in PS and RS. (H) Schematic diagram of ZCCHC8 regulating young L1 silencing during spermatogenesis. ZCCHC8 directly targeted L1 transcripts and influenced its RNA level. Histone modifications of H3K9me3 and H3K4me3 were regulated at young L1 promoters in SSC and PS, which may have impacted the chromatin accessibility of L1Md_A in PS and RS. Data in (A) and (D) are presented as the mean ± SEM of biological replicates. Unpaired one-tailed Student's *t*-test was used to calculate the *P*-values in (A) and (D). **P < 0.01; ***P < 0.001. Two-tailed unpaired Wilcoxon test was used to calculate the *P*-values in (C) and (F).

High levels of H3K4me3 may cause issues with chromatin condensation and the abundant presence of L1 RNA is also associated with higher chromatin accessibility [24]. We suspect that the problems exist with chromatin condensation in KO cells during post-meiotic divisions from PS to haploid RS. We employed transposase-accessible chromatin sequencing (ATAC-seq) to analyse chromatin accessibility in the PS and RS from adult testes. The results showed a significant increase in chromatin accessibility, with a marked peak increase in the L1 region at the PS stage (Fig. 5A). Chromatin accessibility in the promoter regions of focused L1Md_A is notably increased in KO PS (Fig. 5B and C). Similarly to PS, in sorted RS, the total ATAC peak number was increased and the average L1Md_A signal was significantly higher in KO cells (Fig. 5D–G). This preferential effect may be related to differences in the promoter sequences and RNA structures, and the evolutionary ages of A-type L1 compared with other subfamilies of L1 [33,34]. The defects in chromatin condensation may be a significant contributing factor to the decreased meiotic efficiency that is triggered by ZCCHC8 and the obstacles to sperm maturation.

## DISCUSSION

In this work, we demonstrated that ZCCHC8 directly binds to the young L1 RNA during spermatogenesis, influencing their RNA levels and chromatin states at genomic loci. ZCCHC8 depletion led to a reduction in H3K9me3 modifications and a rise in RNA levels in L1 regions in SSC, resulting in a decrease in SSC populations and a postponement of the first wave of spermatogenesis. Furthermore, the absence of ZCCHC8 was associated with an elevation in H3K4me3 and chromatin accessibility at L1Md_A sites during the pachytene stage of meiosis, which may contribute to issues with chromatin condensation and a reduction in spermatids production (Fig. 5H).

Prior research has predominantly focused on the epigenetic silencing factors of transposable elements in the early stage of male germ cells, including small non-coding piRNAs, DNA methylation and repressed H3K9me2/3 modifications. The piRNA system destroys retrotransposon-derived RNAs and guides de novo DNA methylation at some TE promoters. When cells progress to spermatocytes, DNA methylation is the main repressive epigenetic factor for TE silencing, as evidenced by DNMT3L mutant mice [10,14,15,35]. In this context, our study introduces a novel perspective by identifying the role of ZCCHC8—a component of the NEXT complex—in L1 RNA degradation via the RNA exosome pathway in both SSC and spermatocytes. This discovery broadens our understanding of the molecular mechanisms that govern retrotransposon regulation during male germ-cell development.

Unlike the defects of de novo DNA methylation triggered by piRNA abnormalities in fetal germ cells, the knockout of ZCCHC8 impacts H3K9me3 in the L1 regions in postnatal SSC, as well as H3K4me3 during the meiotic stages especially in PS. In this process, the production of piRNAs displays no notable disparities. This suggests that the effect of ZCCHC8 on epigenetic alterations in L1 regions distinctly diverges from the mechanisms that was previously understood to be governed by piRNAs. In studies on embryonic stem (ES) cells, ZCCHC8 has been found to participate in the degradation of chromatin-associated RNAs, including L1 RNA, potentially affecting the expression activity of genes adjacent to L1 encoding. Mechanistically, the accumulation of chromatin-associated RNAs (carRNAs) on chromatin is thought to promote the recruitment of active transcriptional signals such as H3K4me3/H3K27ac and the YY1-EP300 complex [29,30,36–38]. The HUSH complex member MPP8 might interact with ZCCHC8 to participate in the regulation of H3K9me3 modification at L1 loci [25]. In SSC and during meiosis, the knockout of ZCCHC8 significantly affects the histone modifications and chromatin accessibility of L1 loci, with changes often more pronounced than in ES cells. These results indicate that ZCCHC8-mediated epigenetic regulation on TE chromatin plays an irreplaceable role during meiotic progression. Whether ZCCHC8 regulates the L1 chromatin via L1 carRNA occupancy is worth further contemplation and exploration. Our work suggests that, in differential regulatory models, the RNA surveillance system that mediates RNA–chromatin crosstalk may play a significant role.

## METHODS

The detailed methods and materials are available as Supplementary Data at NSR online.

## Supplementary Material

nwae407_Supplemental_Files
