# Supplementary material for: Core factor of NEXT complex, ZCCHC8, governs the silencing of LINE1 during spermatogenesis
Source: Natl Sci Rev. 2024 Dec 17;12(1):nwae407. doi: 10.1093/nsr/nwae407 (PMC11697976; doi:10.1093/nsr/nwae407)
Supplement: nwae407_Supplemental_Files [file nwae407_supplemental_files.zip › supplement figure.pdf]

Sup1

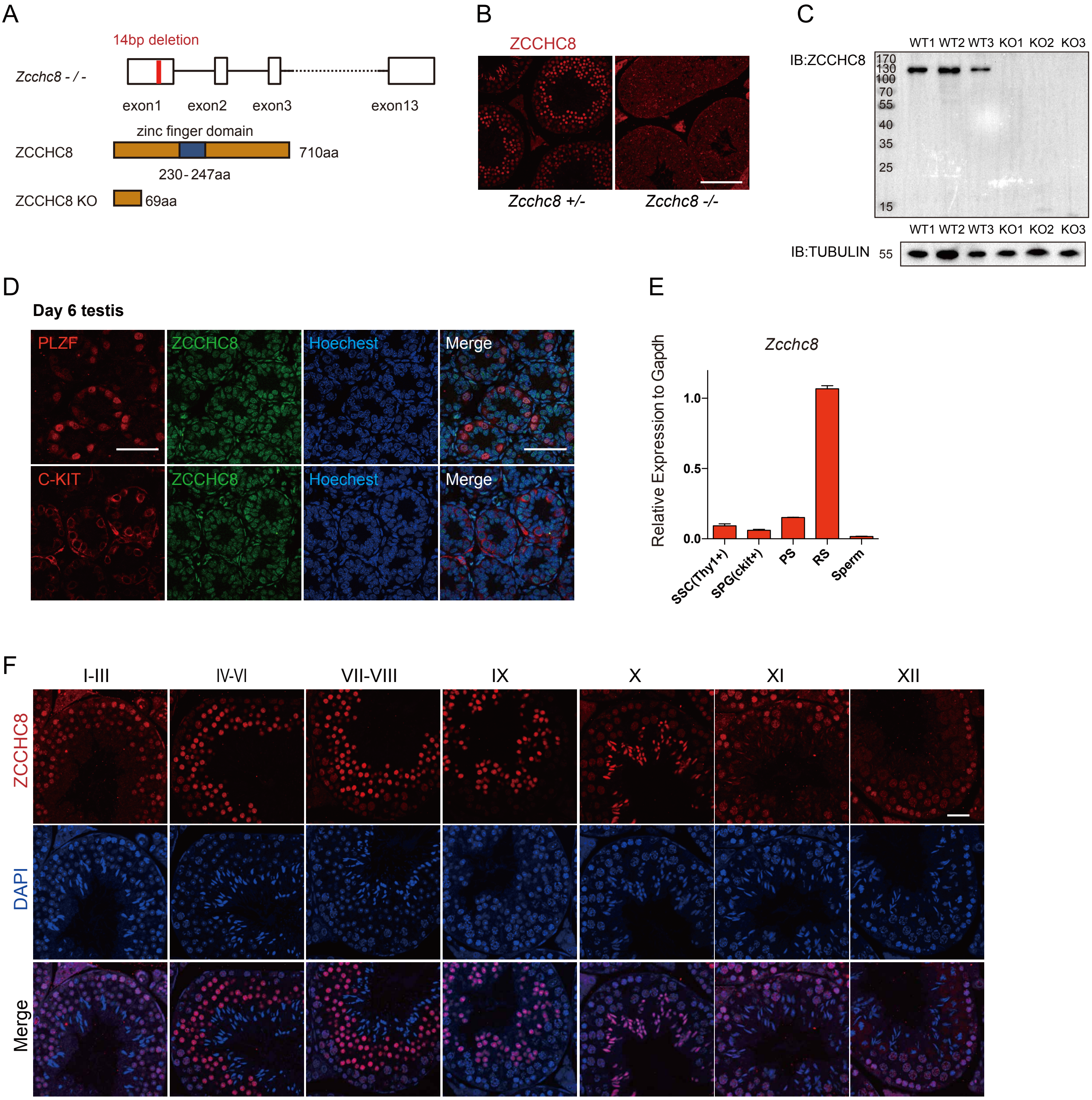
A
14bp deletion
Zcchc8 - / -
exon1
exon2
exon3
exon13
zinc finger domain
ZCCHC8
710aa
230-247aa
ZCCHC8 KO
69aa
B
ZCCHC8
Zcchc8 +/-
Zcchc8 -/-
C
WT1 WT2 WT3 KO1 KO2 KO3
IB:ZCCHC8
170
130
100
70
55
40
35
25
15
IB:TUBULIN
55
D
Day 6 testis
PLZF
ZCCHC8
Hoechest
Merge
C-KIT
E
Zcchc8
Relative Expression to Gapdh
1.0
0.5
0.0
SSC(Thy1+)
SPG(ckit+)
PS
RS
Sperm
F
I-III
IV-VI
VII-VIII
IX
X
XI
XII
DAPI

Supplemental Figure Legends

Figure S1. Relative to Fig.1. Expression pattern of ZCCHC8 during spermatogenesis.

A. Schematic diagram of *Zcchc8* KO mice construction.
B. Immunostaining of ZCCHC8 in WT and *Zcchc8* KO adult testes. Scale bar=50 μm.
C. Western blot of ZCCHC8 and TUBULIN in WT and *Zcchc8* KO adult testes.
D. Localization of ZCCHC8, PLZF, C-KIT in testes at P6. Scale bar=50 μm.
E. Relative expression level of *Zcchc8* in different type of spermatogonial stem cell(SSC), spermatogonia(SPG), pachytene spermatocytes(PS), round spermatids(RS) and sperm. Data are presented as the mean ± SD of 3 technical replicates.
F. Localization of ZCCHC8 in adult testis of different spermatogenic stage. Scale bar=50 μm.

Sup2

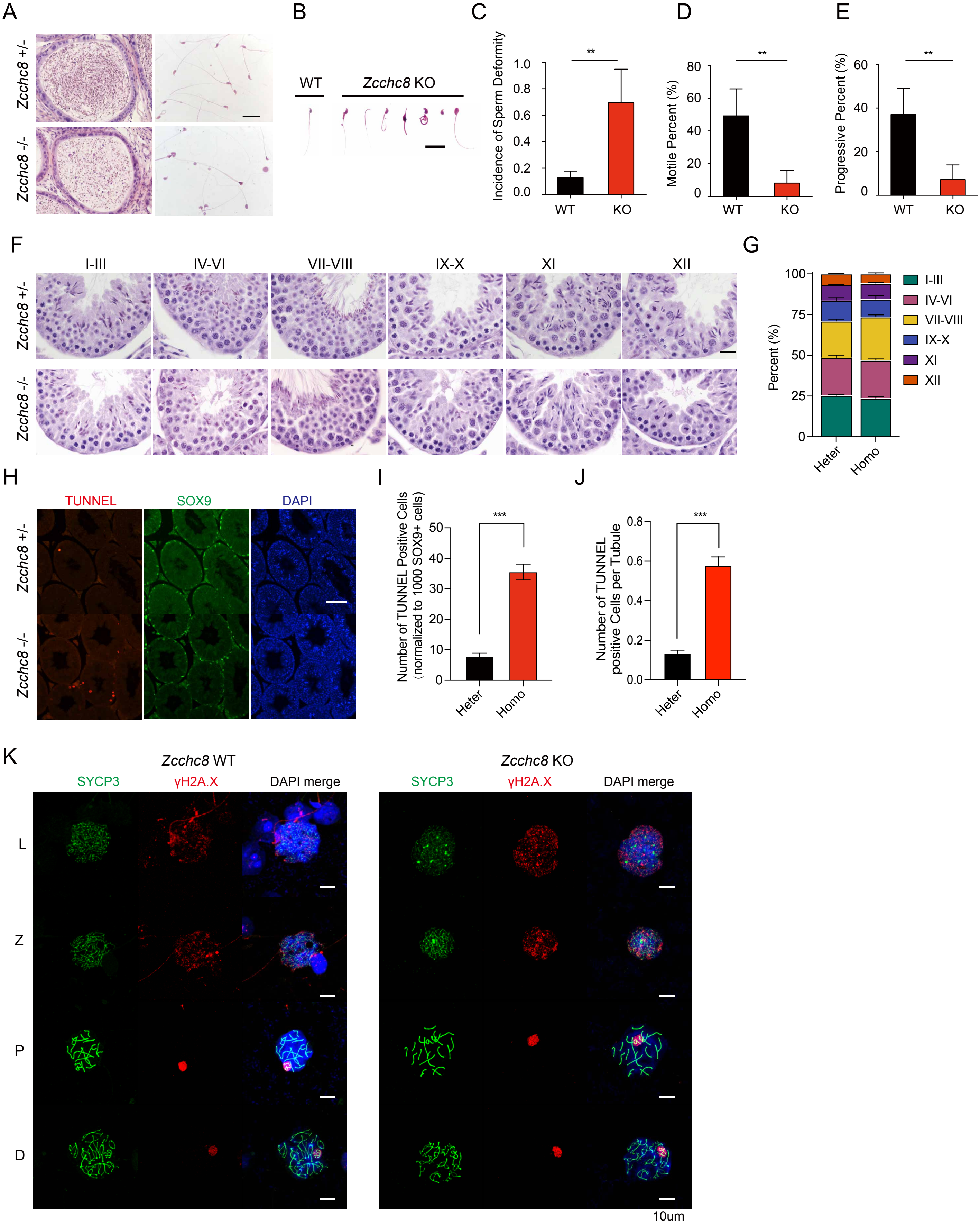

A
Zcchc8 +/-
Zcchc8 -/-
B
WT
Zcchc8 KO
C
Incidence of Sperm Deformity
1.0
0.8
0.6
0.4
0.2
0.0
**
WT
KO
D
Motile Percent (%)
80
60
40
20
0
**
WT
KO
E
Progressive Percent (%)
60
40
20
0
**
WT
KO
F
I-III
IV-VI
VII-VIII
IX-X
XI
XII
Zcchc8 +/-
Zcchc8 -/-
G
Percent (%)
100
75
50
25
0
Heter
Homo
I-III
IV-VI
VII-VIII
IX-X
XI
XII
H
TUNNEL
SOX9
DAPI
Zcchc8 +/-
Zcchc8 -/-
I
Number of TUNNEL Positive Cells (normalized to 1000 SOX9+ cells)
50
40
30
20
10
0
***
Heter
Homo
J
Number of TUNNEL positive Cells per Tubule
0.8
0.6
0.4
0.2
0.0
***
Heter
Homo
K
Zcchc8 WT
Zcchc8 KO
SYCP3
γH2A.X
DAPI merge
L
Z
P
D
10um

Figure S2. Relative to Fig.1. Defects of spermatids and spermatocytes in *Zcchc8* KO mice.

A. Hematoxylin and eosin-stained testis sections showing epididymis and sperm morphology in WT and *Zcchc8* KO mice. scale bar=20 μm.

B. H&E staining of sperm from WT and *Zcchc8* KO adult mice.

C-E. CASA assay of sperm deformity (C), motility (D), and progressive percentage (E) from *Zcchc8* control and KO adult mice. N=5.

F. Periodic acid-Schiff staining showing normal manchette morphology in WT and *Zcchc8* KO testes. scale bar=20 μm.

G. Percentage of spermatocytes calculated through Periodic acid-Schiff staining in control and *Zcchc8* KO mice. N=3.

H. TUNNEL staining of WT and *Zcchc8* KO adult testis. scale bar=50 μm.

I. Statistical analysis of TUNNEL signal normalizing to 1000 SOX9+ cells. N=3.

J. Statistical analysis of TUNNEL signal normalizing by tubule numbers. N=3.

K. Chromatin spread of spermatocytes and immunostaining of SYCP3 and γH2A.X.

Data in C-E,G,I and J are presented as the mean ± SEM of biological replicates. Unpaired one-tailed Student's t test was used to calculate the *P* values in C-E,G,I and J , $^{**}p<0.01$ and $^{***}p< 0.001$.

Sup3

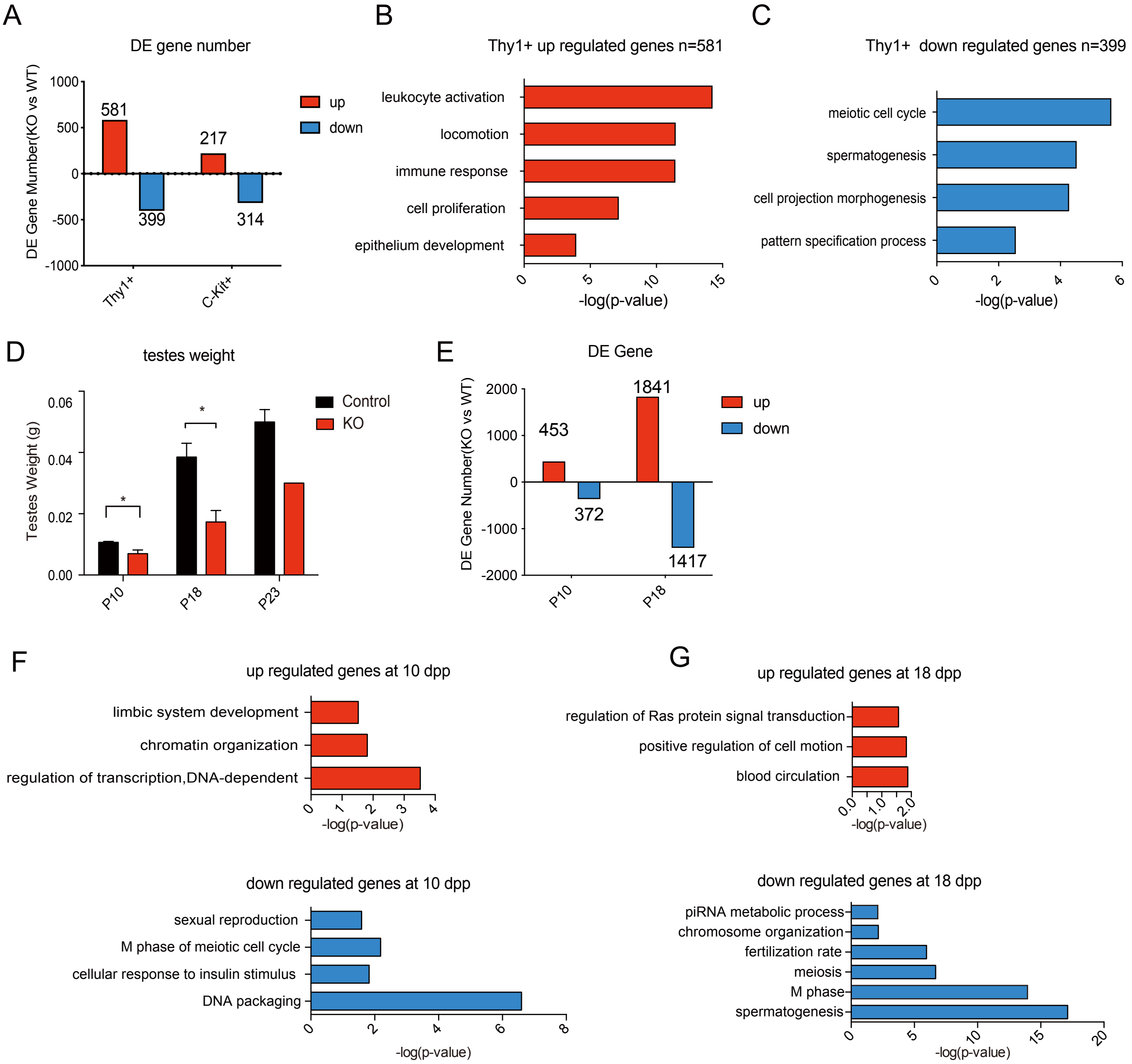
A
DE gene number
DE Gene Mumber(KO vs WT)
1000
500
0
-500
-1000
581
399
217
314
up
down
Thy1+
C-Kit+
B
Thy1+ up regulated genes n=581
leukocyte activation
locomotion
immune response
cell proliferation
epithelium development
0
5
10
15
-log(p-value)
C
Thy1+ down regulated genes n=399
meiotic cell cycle
spermatogenesis
cell projection morphogenesis
pattern specification process
0
2
4
6
-log(p-value)
D
testes weight
Testes Weight (g)
0.06
0.04
0.02
0.00
*
*
Control
KO
P10
P18
P23
E
DE Gene
DE Gene Number(KO vs WT)
2000
1000
0
-1000
-2000
453
372
1841
1417
up
down
P10
P18
F
up regulated genes at 10 dpp
limbic system development
chromatin organization
regulation of transcription,DNA-dependent
0
1
2
3
4
-log(p-value)
down regulated genes at 10 dpp
sexual reproduction
M phase of meiotic cell cycle
cellular response to insulin stimulus
DNA packaging
0
2
4
6
8
-log(p-value)
G
up regulated genes at 18 dpp
regulation of Ras protein signal transduction
positive regulation of cell motion
blood circulation
0.0
1.0
2.0
-log(p-value)
down regulated genes at 18 dpp
piRNA metabolic process
chromosome organization
fertilization rate
meiosis
M phase
spermatogenesis
0
5
10
15
20
-log(p-value)

Figure S3. Relative to Fig.2. Differential expressed genes in SSC and spermatocytes.

A. Differential expressed (DE) gene number of Thy1+ and c-Kit+ cells from P6 *Zcchc8* Control and KO testes. DE genes are fold change (KO/WT) >1.5 or <-1.5 & FDR<0.05.

B,C. Gene ontology (GO) analysis of up regulated genes (B) and down regulated genes (C) in Thy1+ SSC.

D. Testes weight of P10, P18 and P23 *Zcchc8* control and KO mice. Data are presented as the mean ± SEM of biological replicates. N=3 (except for P23 KO N=1), *, $p < 0.05$, (unpaired one-tailed Student's t test).

E. Differential expressed (DE) gene number of *Zcchc8* Control and KO testes at P10 and P18. DE genes are expression level fold change (KO/WT) >1.5 or <-1.5 & FDR<0.05.

F,G. Gene ontology (GO) analysis of up regulated genes and down regulated genes at P10 (F) and P18 (G).

Sup4

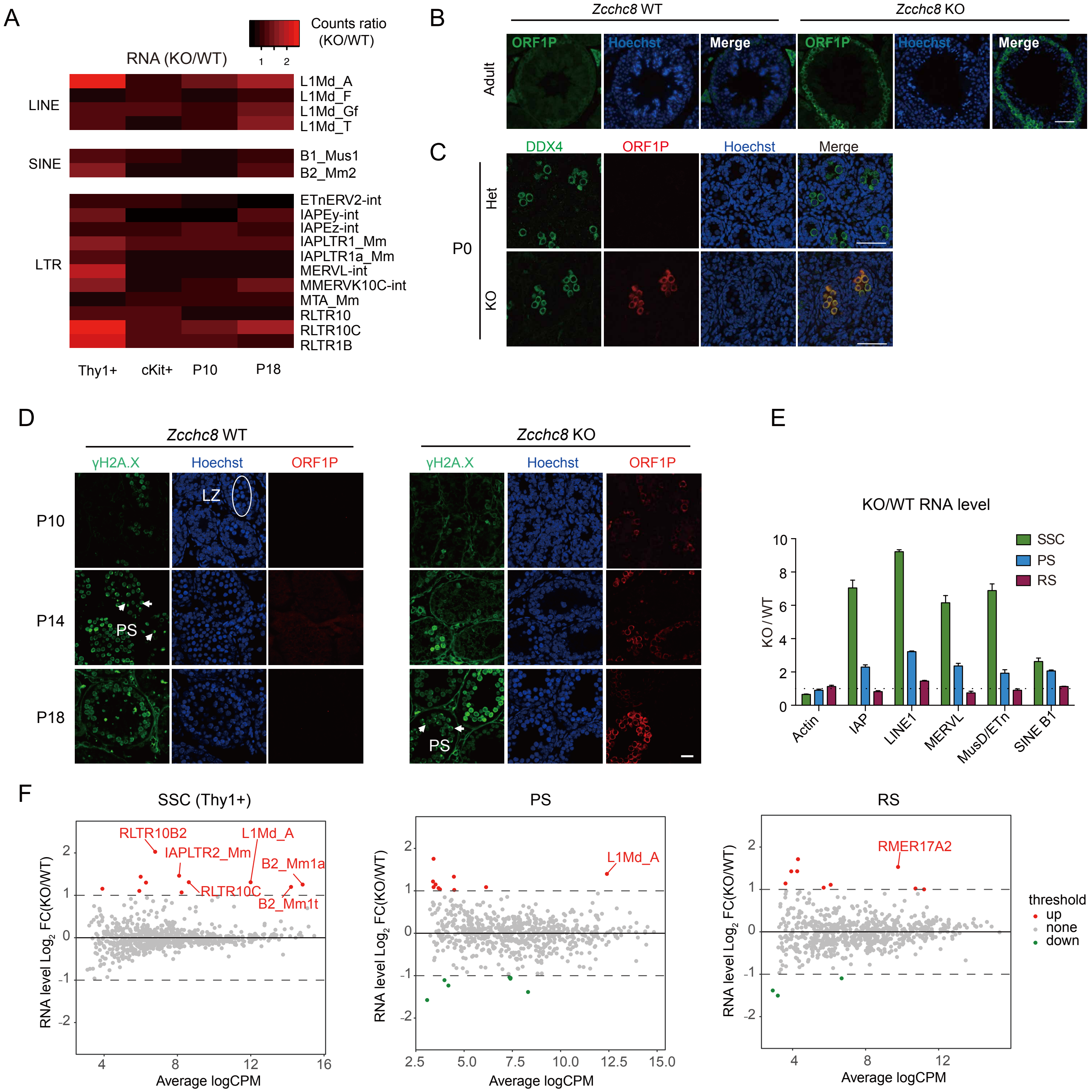

A
RNA (KO/WT)
Counts ratio (KO/WT)
1 2
LINE
SINE
LTR
L1Md_A
L1Md_F
L1Md_Gf
L1Md_T
B1_Mus1
B2_Mm2
ETnERV2-int
IAPEy-int
IAPEz-int
IAPLTR1_Mm
IAPLTR1a_Mm
MERVL-int
MMERVK10C-int
MTA_Mm
RLTR10
RLTR10C
RLTR1B
Thy1+
cKit+
P10
P18
B
Zcchc8 WT
Zcchc8 KO
Adult
ORF1P
Hoechst
Merge
C
DDX4
ORF1P
Hoechst
Merge
P0
Het
KO
D
Zcchc8 WT
Zcchc8 KO
γH2A.X
Hoechst
ORF1P
P10
P14
P18
LZ
PS
E
KO/WT RNA level
SSC
PS
RS
KO / WT
0
2
4
6
8
10
Actin
IAP
LINE1
MERVL
MusD/ETn
SINE B1
F
SSC (Thy1+)
PS
RS
RNA level Log2 FC(KO/WT)
Average logCPM
RLTR10B2
L1Md_A
IAPLTR2_Mm
B2_Mm1a
RLTR10C
B2_Mm1t
RMER17A2
threshold
up
none
down

Figure S4. Relative to Fig.3. Over-activation of L1 during the first wave of spermatogenesis.

A. Heatmap showed RNA fold change of KO/WT SSC, P10 and P18. Representative and high expressed repeat subfamilies are listed.
B. Immunostaining of ORF1P (antibody: Abcam, ab216324) in adult testes of WT and *Zcchc8* KO mice. Positive ORF1P cells are PS cells. Scale bar=50 μm.
C. Immunostaining of DDX4 and ORF1P (homemade) in P0 testes of *Zcchc8* control and KO mice. scale bar=50 μm.
D. Immunostaining of γ H2A.X and ORF1P (homemade) in P10, P14, P18 testes of *Zcchc8* control and KO mice. scale bar=20 μm.
E. KO/WT RNA level of representative repeats tested by RT-qPCR. Dotted line represents fold change=1. Data are presented as the mean ± SD of 3 technical replicates.
F. Plots showing differential expressed repeats. Red plots represent up regulated repetitive subfamilies in KO SSC, PS and RS. Data are presented as the mean of 2 biological replicates.

Sup5

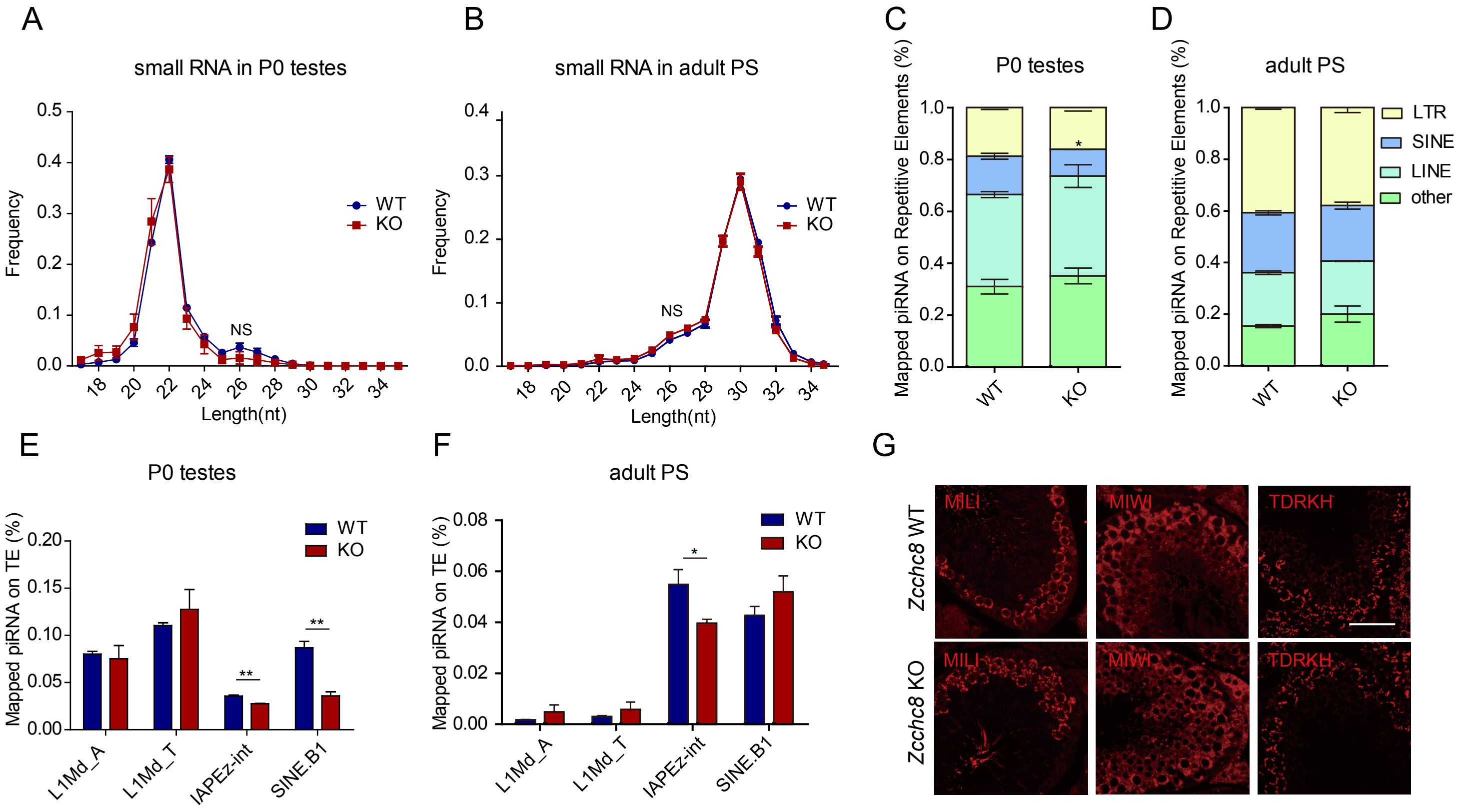

A
small RNA in P0 testes
Frequency
Length(nt)
NS
WT
KO
B
small RNA in adult PS
Frequency
Length(nt)
NS
WT
KO
C
P0 testes
Mapped piRNA on Repetitive Elements (%)
WT
KO
D
adult PS
Mapped piRNA on Repetitive Elements (%)
LTR
SINE
LINE
other
WT
KO
E
P0 testes
Mapped piRNA on TE (%)
WT
KO
L1Md_A
L1Md_T
IAPEz-int
SINE.B1
F
adult PS
Mapped piRNA on TE (%)
WT
KO
L1Md_A
L1Md_T
IAPEz-int
SINE.B1
G
Zcchc8 WT
Zcchc8 KO
MILI
MIWI
TDRKH

Figure S5. Relative to Fig.3. piRNA analysis in SSC and PS.

A． Nucleotide (nt) length distribution of small RNAs in WT (N=3) and KO (N=2) P0 testes.

B． Nucleotide (nt) length distribution of small RNAs in WT (N=4) and KO (N=3) adult PS.

C． Annotation of piRNAs mapping to the genome in P0 testes and PS.

D． Annotation of piRNAs mapping to the genome in P0 testes and PS.

E． Relative frequency of piRNAs mapping to L1Md_A, L1Md_T, IAPz_int and SINE B1 in WT and KO P0 testes.

F． Relative frequency of piRNAs mapping to L1Md_A, L1Md_T, IAPz_int and SINE B1 in WT and KO adult PS.

G． Immunostaining of MILI, MIWI and TDRKH in adult testes of *Zcchc8* control and KO mice. scale bar=50 μm.

Data in A-F are presented as the mean ± SEM of biological replicates. Unpaired one-tailed Student's t test was used to calculate the *P* values in A-F , *p<0.05 and **p< 0.01. NS, not significant.

Sup6

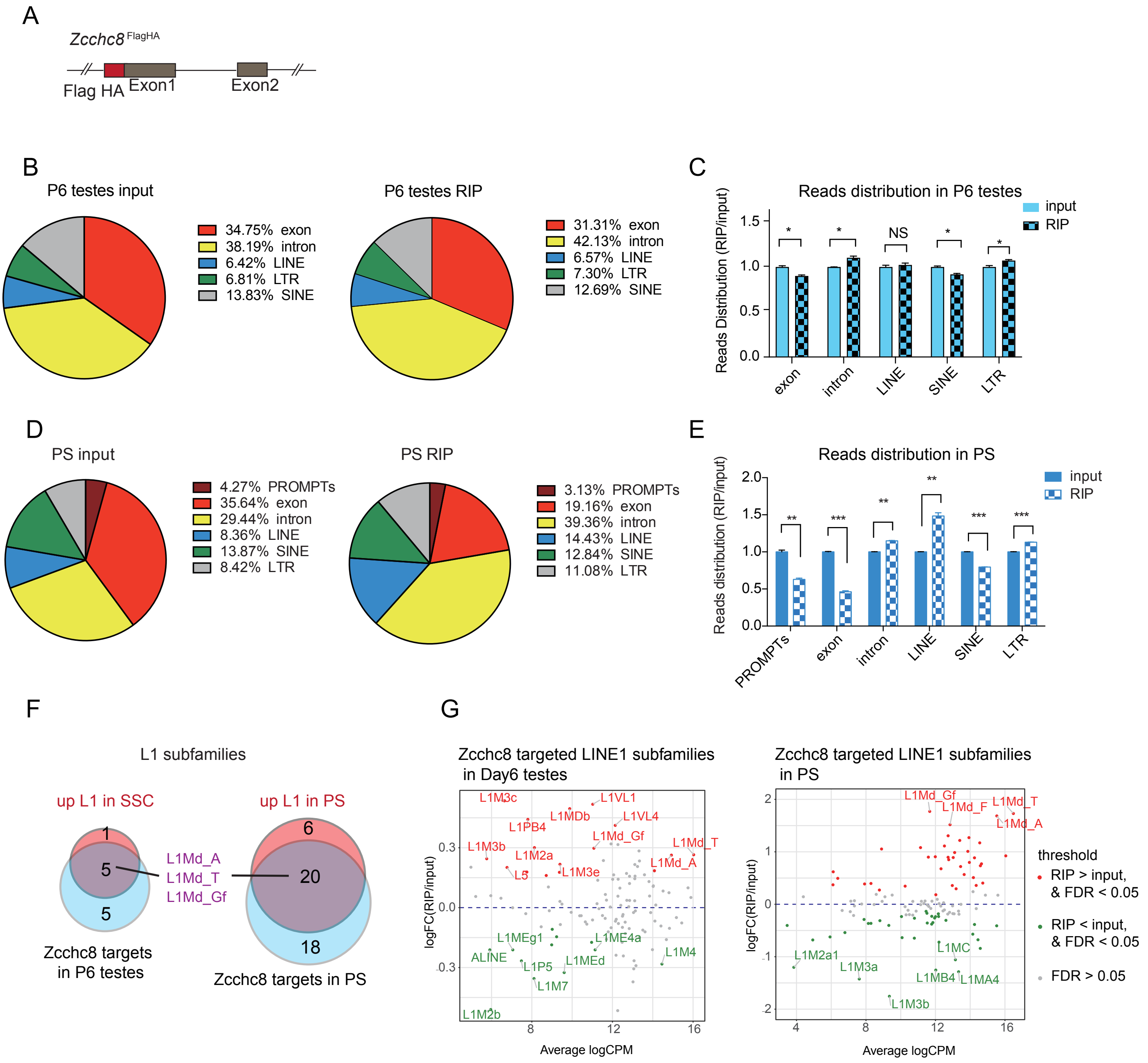
A
Zcchc8 FlagHA
Flag HA
Exon1
Exon2
B
P6 testes input
34.75% exon
38.19% intron
6.42% LINE
6.81% LTR
13.83% SINE
P6 testes RIP
31.31% exon
42.13% intron
6.57% LINE
7.30% LTR
12.69% SINE
C
Reads distribution in P6 testes
input
RIP
Reads Distribution (RIP/input)
NS
exon
intron
LINE
SINE
LTR
D
PS input
4.27% PROMPTs
35.64% exon
29.44% intron
8.36% LINE
13.87% SINE
8.42% LTR
PS RIP
3.13% PROMPTs
19.16% exon
39.36% intron
14.43% LINE
12.84% SINE
11.08% LTR
E
Reads distribution in PS
input
RIP
Reads distribution (RIP/input)
PROMPTs
exon
intron
LINE
SINE
LTR
F
L1 subfamilies
up L1 in SSC
up L1 in PS
L1Md_A
L1Md_T
L1Md_Gf
Zcchc8 targets in P6 testes
Zcchc8 targets in PS
G
Zcchc8 targeted LINE1 subfamilies in Day6 testes
logFC(RIP/input)
Average logCPM
Zcchc8 targeted LINE1 subfamilies in PS
logFC(RIP/input)
Average logCPM
threshold
RIP > input, & FDR < 0.05
RIP < input, & FDR < 0.05
FDR > 0.05

Figure S6. Relative to Fig.3. ZCCHC8 targeted young L1 in SSC and PS.

A. Schematic diagram of Flag-HA-Zcchc8 mice.
B. Pie plot showing ratio of mapped reads in different genome sites in input and RIP samples of P6 testes. Data are presented as the mean of 2 biological replicates.
C. Relative reads distribution of input and RIP in P6 testes. Data are presented as the mean ± SEM of two biological replicates. *$p < 0.05$ (unpaired one-tailed Student's t test).
D. Pie plot showing ratio of mapped reads in different genome sites in input and RIP samples of adult PS. Data are presented as the mean of 2 biological replicates.
E. Relative reads distribution of input and RIP in adult PS. Data are presented as the mean ± SEM of two biological replicates. **$p < 0.01$, ***$p < 0.001$ (unpaired one-tailed Student's t test).
F. Venn plots showing overlapping number up regulated LINE1 subfamilies and RIP targets in P6 SSC and adult PS.
G. Zcchc8 targeted LINE1 subfamilies in P6 testes and adult PS. Data are presented as the mean of 2 biological replicates.

Sup7

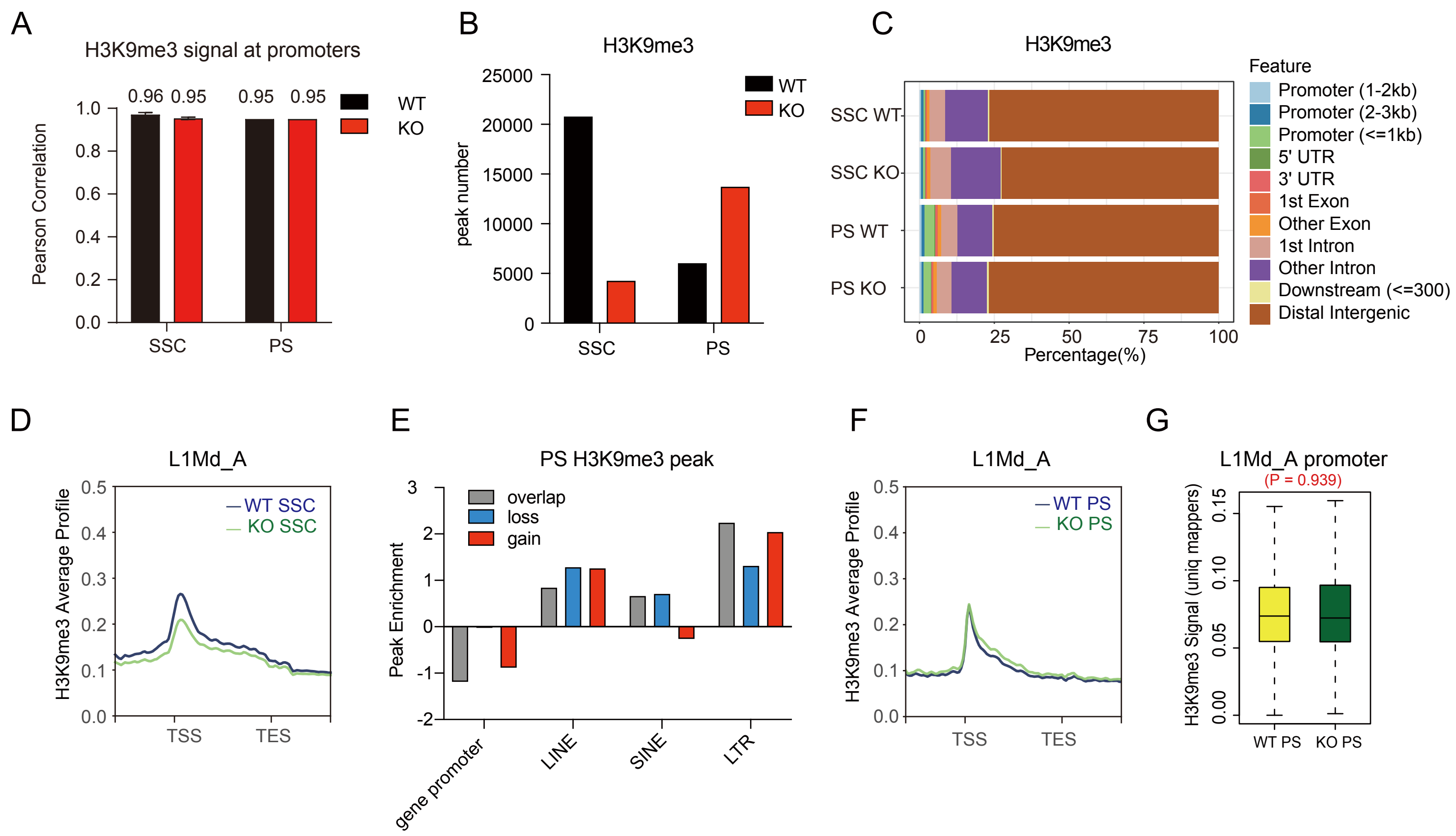
A
H3K9me3 signal at promoters
Pearson Correlation
0.96 0.95 0.95 0.95
WT
KO
SSC
PS
B
H3K9me3
peak number
WT
KO
SSC
PS
C
H3K9me3
SSC WT
SSC KO
PS WT
PS KO
Percentage(%)
Feature
Promoter (1-2kb)
Promoter (2-3kb)
Promoter (<=1kb)
5' UTR
3' UTR
1st Exon
Other Exon
1st Intron
Other Intron
Downstream (<=300)
Distal Intergenic
D
L1Md_A
H3K9me3 Average Profile
WT SSC
KO SSC
TSS
TES
E
PS H3K9me3 peak
Peak Enrichment
overlap
loss
gain
gene promoter
LINE
SINE
LTR
F
L1Md_A
H3K9me3 Average Profile
WT PS
KO PS
TSS
TES
G
L1Md_A promoter
(P = 0.939)
H3K9me3 Signal (uniq mappers)
WT PS
KO PS

Figure S7. Relative to Fig.4. ZCCHC8 deletion results in H3K9me3 loss on young L1 elements.

A. Pearson correlation between replicates for H3K9me3 signal at promoters in SSC (N=3) and PS (N=2). Data are presented as the mean ± SEM of biological replicates.
B. Peak number of H3K9me3 in SSC and PS. Data are presented using pooled samples with 3 or 2 replicates (See methods).
C. H3K9me3 peak distribution in different genomic regions. The *P* value is calculated by the chi-square test. *P* value in SSC is 6.514795e-07, and in PS is 0.0007040506.
D. Average signal profile of H3K9me3 at promoter of L1Md_A in SSC.
E. H3K9me3 peak enrichment of gain, loss and overlapping peaks in different genomic regions in PS.
F. Average signal profile of H3K9me3 at promoter of L1Md_A in PS.
G. Boxplot showing H3K9me3 signal intensity at promoter regions (-3kb to +3kb of TSS) of intact L1Md_A (L1Md_A length > 3kb, n=3958) loci in PS, calculated using unique mappers. P = *P* value calculated by Wilcoxon test, two-sided.

Sup8

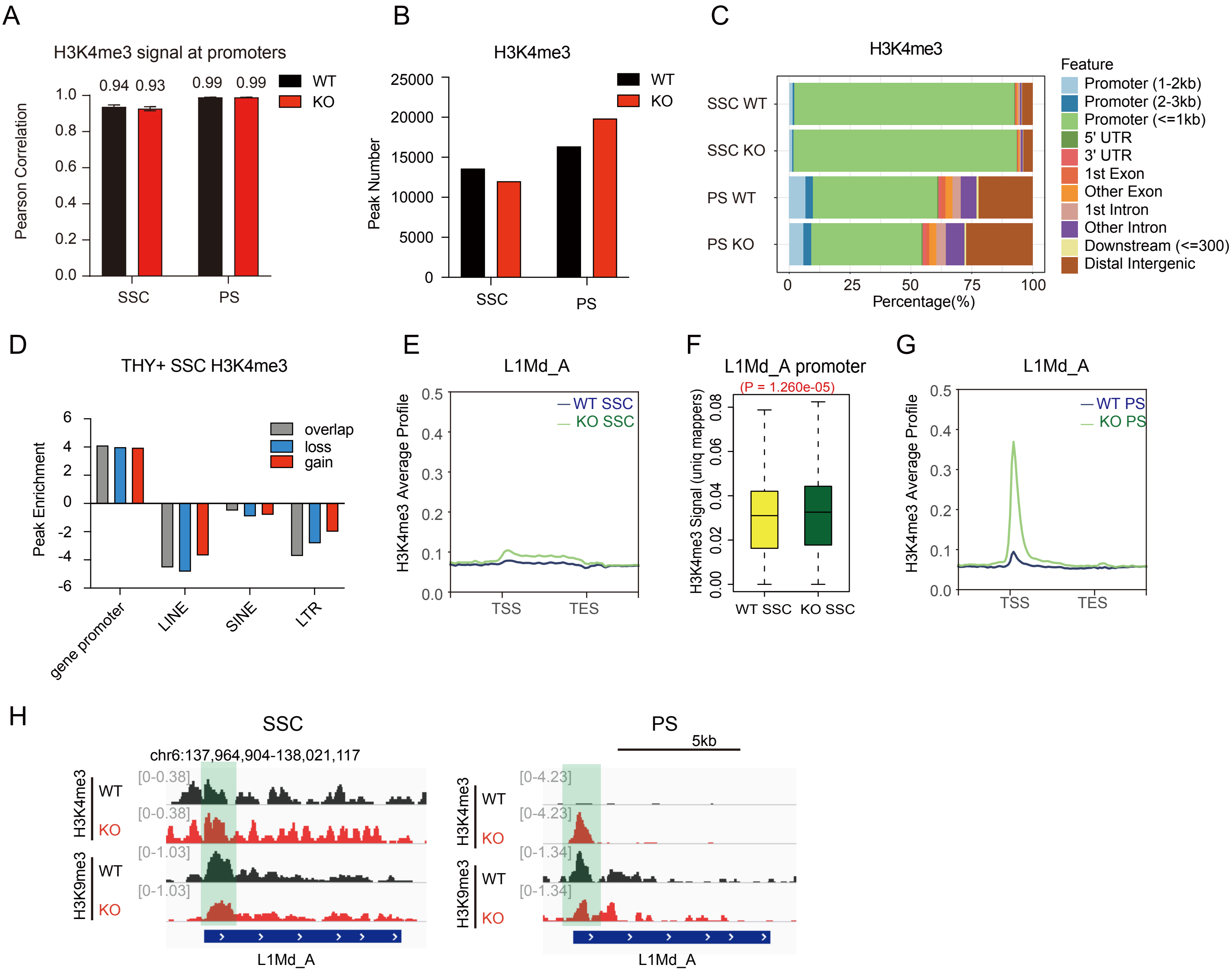

A
H3K4me3 signal at promoters
Pearson Correlation
0.94 0.93 0.99 0.99
WT
KO
SSC
PS
B
H3K4me3
Peak Number
WT
KO
SSC
PS
C
H3K4me3
SSC WT
SSC KO
PS WT
PS KO
Percentage(%)
Feature
Promoter (1-2kb)
Promoter (2-3kb)
Promoter (<=1kb)
5' UTR
3' UTR
1st Exon
Other Exon
1st Intron
Other Intron
Downstream (<=300)
Distal Intergenic
D
THY+ SSC H3K4me3
Peak Enrichment
overlap
loss
gain
gene promoter
LINE
SINE
LTR
E
L1Md_A
H3K4me3 Average Profile
WT SSC
KO SSC
TSS
TES
F
L1Md_A promoter
(P = 1.260e-05)
H3K4me3 Signal (uniq mappers)
WT SSC
KO SSC
G
L1Md_A
H3K4me3 Average Profile
WT PS
KO PS
TSS
TES
H
SSC
chr6:137,964,904-138,021,117
H3K4me3
H3K9me3
WT
KO
[0-0.38]
[0-1.03]
L1Md_A
PS
5kb
[0-4.23]
[0-1.34]
L1Md_A

Figure S8. Relative to Fig.4. ZCCHC8 deletion results in increase of H3K4me3 on young L1 elements.

A. Pearson correlation between replicates for H3K4me3 signal at promoters in SSC (N=3) and PS (N=3).

B. Peak number of H3K4me3 in SSC and PS. Data are presented using pooled samples with 3 replicates (See methods).

C. H3K4me3 peak distribution in different genomic regions. The *P* value is calculated by the chi-square test. *P* value in SSC is 0.1593419, and in PS is 1.149818e-37.

D. H3K4me3 peak enrichment of gain, loss and overlapping peaks in different genomic regions in SSC.

E. Average signal profile of H3K4me3 at promoter of L1Md_A in SSC.

F. Boxplot showing H3K4me3 signal intensity at promoter regions (-3kb to +3kb of TSS) of intact L1Md_A (L1Md_A length > 3kb, n=3958) loci in SSC, calculated using unique mappers. P = *P* value calculated by Wilcoxon test, two-sided.

G. Average signal profile of H3K4me3 at promoter of L1Md_A in PS.

H. Genome browser track showing H3K9me3 and H3K4me3 signal of L1Md_A locus in SSC and PS.
