# Supplementary material for: Core factor of NEXT complex, ZCCHC8, governs the silencing of LINE1 during spermatogenesis
Source: Natl Sci Rev. 2024 Dec 17;12(1):nwae407. doi: 10.1093/nsr/nwae407 (PMC11697976; doi:10.1093/nsr/nwae407)
Supplement: nwae407_Supplemental_Files [file nwae407_supplemental_files.zip › supplemental_file.pdf]

Methods

Generation of Zcchc8-KO and Flag-HA-Zcchc8 mice

Zcchc8-KO mice were previously reported [1]. Flag-HA knock-in mice (FH-Zcchc8) were generated using CRSPR-cas9 system. Flag-HA single strand DNA(ssDNA) donor of 137nt (see supplemental table 4) was synthetized and injected to zygotes together with sgRNA at a final concentration of 1 μM. Gene edited mice were mated back with WT C57BL/6 mice for several generations.

Histological and immunofluorescent staining analysis

Haematoxylin and eosin (H&E) staining and immunofluorescent staining of testes were described previously [2]. Primary antibodies used in this study were as follows: anti-ZCCHC8 (Proteintech, 23374), anti-γH2A.X (Abcam, ab26350), anti-PLZF (R&D, AF2944), anti ORF1P (Abcam, ab216324 and homemade antibody gifts from Prof. Ramesh Pillai [3]), anti-c-KIT (R&D, AF1356), anti-DDX4 (Abcam, ab13840), anti-MILI (MBL, PM044), anti-MIWI (Cell Signaling Technology, 2079), anti-TDRKH (Proteintech, 13528-1-AP) and PNA (Sigma, L7381). TUNEL assay was performed with TUNEL BrightRed Apoptosis Detection Kit (Vazyme, A113-01) according to the manufacturer's instructions.

Chromosome spread and immunofluorescence analysis

Spermatocyte spreads were prepared as the following steps. Seminiferous tubules were collected and shreded, treated with 0.5% NaCl at room temperature for 10 minutes. Take the 10 μL cell suspension to 100 μL 1% PFA with 0.15% Triton X-100, and spread the cells on a glass slide in a wet box overnight. Slides were washed with TBS several times, and blocked with 3% BSA at room temperature for 1 h. Primary antibodies used for immunofluorescence were as follows: mouse anti-SYCP3 (1:200 dilution; Abcam ab97672), Anti-phospho-Histone H2A.X (Ser139) Antibody (1:200 dilution; Cell Signaling Technology, 9718S). Primary antibody was incubated overnight at 37 °C and were detected with Alexa Fluor 488- or 594-conjugated secondary antibodies and DAPI for 1 h at 37 °C. After being washed with TBS several times, the slides were mounted using Mounting medium, fluoroshield.

Computer-assisted sperm analysis

Sperm was collected from cauda epididymides and incubated with HTF medium (Irvine Scientific, 90125) for 5 min at 37°C. 10 μL of sperm suspension was placed in slide chamber and analyzed by IVOS II system (Hamilton Thorne) with default parameters.

In vitro fertilization (IVF)

For IVF, sperm was harvested from cauda epididymides of control (*Zcchc8* WT or heterozygous) and *Zcchc8* KO mice aged 10–20 weeks and incubated in IVF-plus medium for 30 min. MII oocytes were collected 16 h after human chorionic gonadotropin injection. IVF was conducted in IVF-PLUS medium for 4h. Excessive sperm were removed after 4h of IVF.

Isolation of spermatogonia

Spermatogonia were isolated from WT and *Zcchc8* KO mice aged 6-8 days. Testes were collected after removing the tunica albuginea membrane. After washing in PBS one time, tubules were cut

into pieces using sterilizing scissors. 1mL pre-heated 0.05% typsin was added to digest the tubules for 5 min at 35°C with shaking and pipetting. 100 μL FBS was added to stop trypsinization. Cells were filtered with a 40 μm nylon cell strainer. After centrifuging cells and removing supernatant, cells were suspended using 500 μL PBS. 50 μL suspension was kept as negative control. 0.3 μL Thy1 and c-Kit FACS antibody was added and incubated for 20 min on ice in dark. Antibody was washed one time with PBS before flow cytometric analysis and fluorescence activated cell sorting (FACS). $Thy1^{+}$ c-kit $^{-}$ ($Thy1^{+}$) cells and c-kit $^{+}$ $thy1^{-}$ (c-kit^{+}) cells were collected.

Isolation of pachytene spermatocytes and round spermatids

Pachytene spermatocytes and round spermatids were isolated *via* flow cytometry as previously described with some modifications [4]. Briefly, after the removal of tunica albuginea, each testis was first digested in 1mg/mL collagenase IV solution (diluted in DMEM) for 5 min at 35 °C with shaking. After settling for 2 min at room temperature (RT), the supernatant was removed. 5 mL pre-heated 0.05% trypsin was added to digest the tubules for about 8min with gently shaking and pipetting at 35°C. The resulting suspension was passed through a 100 μm nylon cell strainer. 50 μL FBS was added for stopping digestion. After spin down cells and removal of supernatant, cells were suspended in 20ml DMEM with 6μg/million cells Hoechst-33342. The suspension was pipetted up and down 10 times and incubated for 20 min at 35°C. Finally, cells were washed with PBS and passed through a 40 μm nylon cell strainer before FACS. Data analysis was done using BD FACS AriaII. Gate settling was done as previously reported [4].

RNA-binding protein immunoprecipitation

0.5-1 million collected cells were washed with PBS one time. Cells were centrifuged at 1000rpm for 5 min. Supernatant was removed and cell pellet was suspended in lysis buffer (50 mM Tris-HCl pH 7.4, 100 mM KCl, 12 mM $MgCl_2$, 1% Triton-X100, 1 mM DTT, 100 μg/mL cycloheximide, protease inhibitor cocktail, 250U/mL RNase inhibitor, 125U/ml SUPERase-in) for 10 min on ice. 10% suspension was kept as input. 10 μL Dynabeads protein A and 10 μl Dynabeads protein G were incubated with 3μg HA-antibody or 3μg igG for 3 hours with rotation at 4°C. Then antibody coated beads were washed three times using lysis buffer before incubated with cell lysis for 4-5 hours with rotation at 4°C. After immunoprecipitation, resulting beads were washed using high salt wash buffer (50 mM Tris-HCl pH 7.4, 300 mM KCl, 12 mM $MgCl_2$, 1% Triton-X100, 1 mM DTT, 100 μg/mL cycloheximide, protease inhibitor cocktail) for 5 times. Beads and input sample were then suspended in RNAiso-plus(Takara) and mix well. 1/5 volume chloroform was added and mix well. Maxtract High Density (Qiagen) was used for RNA extraction. 1/10 volume of 3M NaAc, 1μL glycogen and equal volume of isopropanol were used to precipitate RNA at -20 °C for over 30 min. RNAs were washed in 75% ethanol twice and eluted in DEPC treated water.

Ribosomal RNA-free RNA-seq

The total RNA was extracted from more than 30,000 sorted cells or whole testes using the RNeasy Mini Kit (Qiagen). The total RNA (100ng - 1μg) was used for RNA-seq using the KAPA Stranded RNA-Seq with RiboErase (Kapa, KK8483) following the manufacturer's instructions. Pair-end-150 bp sequencing was further performed on Illumina NovaSeq 6000.

ATAC-seq library preparation and sequencing

The ATAC-seq libraries of sorted germ cells were prepared as previously described [5]. Briefly, samples were lysed in lysis buffer (10 mM Tris-HCl (pH 7.4), 10 mM NaCl, 3 mM MgCl2 and 0.15% NP-40) for 10 min on ice to prepare the nuclei. Immediately after lysis, nuclei were spun at 500g for 5 min to remove the supernatant. Nuclei were then incubated with the Tn5 transposome and tagmentation buffer at 37 °C for 30 min (Vazyme Biotech). After the tagmentation, the stop buffer was added directly into the reaction to end the tagmentation. PCR was performed to amplify the library for 14 cycles using the following PCR conditions: 72 °C for 3 min; 98 °C for 30s; and thermocycling at 98°C for 15s, 60°C for 30s and 72°C for 3min; following by 72 °C 5 min. After the PCR reaction, libraries from 200bp to 700bp were purified from gel extraction before sequencing. Pair-end-150 bp sequencing was further performed on Illumina NovaSeq 6000 at Berry Genomics.

Ultra-Low-input(ULI)-NChIP-seq library preparation and sequencing

For ULI-NChIP-seq, 10000 sorted cells were used per reaction. The procedure was performed as previously described [6]. Briefly, sorted cells were washed in PBS twice and lysed in 20ul Nuclei Extraction buffer (10mM Tris-HCl[pH 8.5], 140mM NaCl, 5mM MgCl2, 0.6% NP40, 1mM PMSF and protease inhibitor cocktail), then add 30ul MNase Master mix (Final concentration: 1x MNase master buffer, 2mM DTT, 5% PEG6000, 2U/ul MNase) and mix well. Proceed at 25°C for 7.5 min. Add 5.5ul 100mM EDTA and 4ul Nuclear Break Buffer (1% Triton X-100, 1% deoxycholate) and rest on ice for 15min. Add 140 ul ChIP buffer (10mM Tris-HCl[pH 8.0], 90mM NaCl, 2mM EDTA, 0.1% Triton X-100, 0.1% deoxycholate, 1mM PMSF) to make 200ul sheared chromatin for each IP. Keep 4ul as input. Antibody was pre-incubated with dynabeads protein A for 2h and washed twice before IP. Sheared chromatin was rotated and incubated with antibody coated beads at 4°C overnight. Wash twice with Low salt wash buffer (20mM Tris-HCl[pH 8.0], 0.1% SDS, 1% Triton X-100, 2mM EDTA, 150mM NaCl) and twice with High salt wahs buffer (20mM Tris-HCl[pH 8.0], 0.1% SDS, 1% Triton X-100, 2mM EDTA, 500mM NaCl). Add 100ul Hot elution buffer (100mM NaHCO3, 1% SDS) to beads and 96ul Elution buffer (10mM Tris-HCl[pH 8.5]) to input. Put the samples in a shaker at 65°C for 1.5-2h. Remove the beads and proceed DNA isolation. The sequence libraries were generated using the KAPA Hyper Prep Kit for the Illumina platform. Pair-end-150 bp sequencing was further performed on Illumina NovaSeq 6000 at Berry Genomics.

Small RNA sequencing and data processing

For each replicate, two fetal testes or sorted pachytene spermatocytes were pooled and RNA was isolated using the TRIzol reagent following the manufacturer's instructions. 1ug total RNA was prepared for Illumina sequencing using the Small RNA Sample Pre Kit according to manufacturer's protocol. 4 ng of each sample were used for the final library pool and sequenced on a HiSeq/MiSeq sequencer (Illumina) in 50-base single-end read mode. Adaptor sequences were removed from 3′ end of the raw fasta files using cutadapt with default settings. The small RNA sequences with a length larger than 17 nt were retained and mapped to the reference mm9 genome, with no mismatches allowed (Bowtie v1.2). The small RNAs were annotated according to information within the piRbase (release v3.0). Mapped piRNA reads (25-30 nt) were categorized according to annotations from mm9 RepeatMasker and using featureCounts.

RNA-seq analysis

rRNA-free RNA sequencing data were trimmed by Trim_galore (version 0.6.4) for adaptor trimming as well as quality control with the parameters --clip_R1 9 --clip_R2 9 –paired. The trimmed reads were then aligned to the mm9 reference genome using TopHat (v 2.1.1) (Trapnell et al., 2009) with the default parameters. The expression level for each sample was quantified by fragments per kilobase of transcript per million fragments mapped (FPKM) using Cufflinks (v 2.2.1). Repeat reference were downloaded from UCSC RepeatMasker. Gene and repeat counts were calculated using the Htseq-count (v 0.6.0) with parameter -s reverse. Principal component analysis was implemented using the R function prcomp. Differential expressed genes or repeats were defined by edgeR (R package). Functional annotation was performed using the Database for Annotation, Visualization and Integrated Discovery (DAVID) Bioinformatics Resource. Gene ontology terms for each function cluster were summarized to a representative term, and p values were plotted to show the significance. Bigwig file of RNA-seq samples were normalized according to sequencing depth using bedtools genomecov function.

RIP-seq analysis

RIP-seq data were trimmed and aligned to the mm9 reference genome the same as the RNA-seq data. Reads distribution was calculated through mapped reads overlapping with different genome regions using bedtools (v 2.20.1). Targets were defined through edgeR (IP vs input). For IP>input and FDR < 0.05 were LINE1 subfamilies targeted by Zcchc8, and others are non-targets.

ULI-NChIP-seq analysis

ChIP-seq reads were trimmed by Trim_galore and aligned to the mouse genome mm9 using bowtie2 (v 2.2.9) with default parameters. Multi-mapped reads were removed by samtools using the following command: samtools view sample.bam | grep "AS:" | grep -v "XS:". PCR duplicates were removed through bedtools and 10 M unique reads for each sample were ramdonly picked for downstream analysis. Replicates were pooled together for peak calling. H3K4me3 peaks were defined by MACS2 (v 2.0.10) with parameters --nomodel --shift=25 --SPMR. H3K9me3 peaks were defined with parameters --broad --nomodel --shift=25 --SPMR. Peak distribution was calculated by ChIPseeker (R package). Profile of ChIP signal was ploted by siteproBW (0.6.7) with parameters --span=3000 --pf-res=10[6, 7].

ATAC-seq analysis

ATAC-seq reads were trimmed by Trim_galore and aligned to the mouse genome mm9 using bowtie2 (v 2.2.9) with default parameters. PCR duplicates and mitochondria reads were removed through bedtools. 10 M uniquely mapped reads for each replicate were ramdonly picked and merged for downstream analysis. Peaks were defined with –keep-dup all –nomodel –shiftsize 25.

QUANTIFICATION AND STATISTICAL ANALYSIS

Values of biological replicates, p values, exact value of n, and statistical tests, are reported in the Figure legends. If not mentioned otherwise in the Figure legend statistical significance (*p < 0.05; **p < 0.01; ***p < 0.001; ns - not significant) was determined using two-tailed Student’s t test as indicated in the Figure legends.

DATA AND CODE AVAILABILITY

The accession number in GSA for the RNA-seq, RIP-seq and N-ChIP-seq data reported in this paper is GSA: GRA012420.

References:

1. Wu Y, Liu W, Chen J *et al.* Nuclear Exosome Targeting Complex Core Factor Zcchc8 Regulates the Degradation of LINE1 RNA in Early Embryos and Embryonic Stem Cells. *Cell Rep*. 2019; **29**(8): 2461-2472 e2466. doi: 10.1016/j.celrep.2019.10.055
2. Qi M, Sun H, Guo Y *et al.* m(6) A reader protein YTHDF2 regulates spermatogenesis by timely clearance of phase-specific transcripts. *Cell Prolif.* 2022; **55**(1): e13164. doi: 10.1111/cpr.13164
3. Zhang Y, Guo R, Cui Y *et al.* An essential role for PNLDC1 in piRNA 3' end trimming and male fertility in mice. *Cell Res*. 2017; **27**(11): 1392-1396. doi: 10.1038/cr.2017.125
4. Gaysinskaya V, Soh IY, van der Heijden GW *et al.* Optimized flow cytometry isolation of murine spermatocytes. *Cytometry A*. 2014; **85**(6): 556-565. doi: 10.1002/cyto.a.22463
5. Wu J, Huang B, Chen H *et al.* The landscape of accessible chromatin in mammalian preimplantation embryos. *Nature*. 2016; **534**(7609): 652-657. doi: 10.1038/nature18606
6. Liu X, Wang C, Liu W *et al.* Distinct features of H3K4me3 and H3K27me3 chromatin domains in pre-implantation embryos. *Nature*. 2016; **537**(7621): 558-562. doi: 10.1038/nature19362
7. Liu B, He Y, Wu X *et al.* Mapping putative enhancers in mouse oocytes and early embryos reveals TCF3/12 as key folliculogenesis regulators. *Nat Cell Biol*. 2024; **26**(6): 962-974. doi: 10.1038/s41556-024-01422-x
